# Crosslinked Polydiallyldimethylammonium Chloride Adsorbent for the Selective Separation of Rhenium Ions from Pregnant Leach Solutions

**DOI:** 10.3390/ma17112737

**Published:** 2024-06-04

**Authors:** Mohammadbagher Fathi, Mehdi Mahmoudian, Richard Diaz Alorro, Mostafa Chegini

**Affiliations:** 1 Centre for Ore Deposit and Earth Sciences (CODES), University of Tasmania, Hobart, TAS 7001, Australia; 2 Department of Mining Engineering, Faculty of Engineering, Urmia University, Urmia 57561-51818, Iran; 3 Department of Nanotechnology, College of Science, Urmia University, Urmia 57561-51818, Iran; m.mahmoudian@urmia.ac.ir; 4 Nanotechnology Research Institute, Urmia University, Urmia 57561-51818, Iran; 5 Western Australian School of Mines: Minerals, Energy and Chemical Engineering, Curtin University, Kalgoorlie, WA 6430, Australia; richard.alorro@curtin.edu.au; 6 Mineral Processing Laboratory Expert, Amirkabir University of Technology, Tehran 15825-4413, Iran; m_chegini60@yahoo.com

**Keywords:** adsorbent, pregnant leach solutions, rhenium, free-radical polymerization (FRP), pre-industrial and industrial conditions

## Abstract

The depletion of valuable mineral reserves has rendered effluents generated from mining and industrial processing activities a promising resource for the production of precious elements. The synthesis and improvement of new adsorbents to extract valuable compounds from industrial wastes and pregnant leach solutions, besides increasing wealth, can play a significant role in reducing environmental concerns. In this work, a new and low-cost adsorbent for the selective extraction of rhenium (perrhenate ions, ReO_4_^−^) was synthesized by the free-radical polymerization (FRP) of a diallyl dimethylammonium chloride monomer (quaternary amine) in the presence of a crosslinker. Various methods were employed to characterize the polymeric adsorbent. The results revealed that the designed polymeric adsorbent had a high surface area and pores with nano-metric dimensions and a pore volume of 6.4 × 10^−3^ cm^3^/g. Four environments—single, binary, multicomponent, and real solutions—were applied to evaluate the adsorbent’s performance in the selective separation of Re. Additionally, these environments were used to understand the behavior of molybdenum ions, the primary competitors of perrhenate ions in the ion exchange process. In competitive conditions, using variations in *q_e_*_,*mix*_/*q_e_*, an antagonism phenomenon (*q_e_*_,*mix*_/*q_e_* < 1) occurred due to the inhibitory effect of surface-adsorbed molybdenum ions on the binding of the perrhenate ions. However, across all conditions, the separation values for Re were higher than those for the other studied elements (Mo, Cu, Fe).

## 1. Introduction

Hydrometallurgical methods provide a solution to extract and recover precious metals from diluted solutions, effluents, and industrial solid residues, serving as a significant response to evolving environmental challenges. This technique has been introduced as a suitable method for reasonable separations in the field of valuing dilute solutions containing precious metals and environmental remediation. Integrating adsorbents into effective hydrometallurgical processing methods has resulted in significant enhancements in recovering solutions containing metal ions. However, in this process, the competition between ions with the same charges for adsorption by an adsorbent can affect its selectivity [1]. Ion exchange resins which has been used empirically for the recycling of metals are a common class of adsorbents [2,3]. Generally, the current resins include crosslinked polystyrene with divinylbenzene [4]. 

The application of commercial ion exchange resins is hindered by challenges such as poor selectivity towards precious metals, complexity in elution, difficulties in regeneration processes, and high costs [5]. Therefore, there is a need for research to develop more cost-effective and efficient alternative adsorbents for separating and recovering target metal ions.

In recent years, there has been a rise in studies aimed at developing metal-selective resins, with researchers investigating various mechanisms to enhance their selectivity [6]. However, the chemical modification of resins with specific functional groups, which exhibit an affinity for metals of a particular type, is one of the most effective mechanisms for achieving selectivity [7]. In this regard, polystyrene ion exchange resins have been modified with different functional groups and used to separate or recover metal ions or complexes. These ion exchangers are derived by implanting definite functional groups within the polymer matrix by chemical bonds or physical adsorption routes [8,9]. It is worth noting that the increased selectivity of an adsorbent material primarily relies on its matrix and structure, which involve chemical coordination bonds. Selecting particular adsorption groups and unique morphological properties within the polymer is vital during synthesis, as it permits the creation of a resin specifically designed for selective and specialized applications [9].

Re is a rare element in the earth that is widely used in high-end technologies such as metallurgy, chemical, and petrochemical industries due to its unique physical and chemical properties [10]. In potential Re-containing resources, this element is often found alongside molybdenum. The similarity in properties, with Re^4+^ and Mo^4+^ having ionic radii of 0.72 Å and 0.70 Å, respectively, poses a significant challenge in the separation and recovery of Re [11]. As a result, in the presence of molybdenum, the extraction of valuable amounts of Re necessitates a selective adsorbent. 

The high affinity of perrhenate anions (ReO_4_^−^) for functionalized resins makes the ion exchange method a promising approach for selective separation from bearing solutions, outperforming other methods such as precipitation and solvent extraction [12]. A variety of resins have been used for Re separation, and they can be reactivated and reused multiple times. Purolite A170 and A172 are widely used anion exchange resins specifically designed for the selective adsorption of rhenium anions, making them ideal for this application [13]. In a recent study led by Bozorov et al., they explored the sorption mechanism of Re from a binary Re-Mo solution using various sorbents, including A-100, A-170, A-172, and BO-020 [13]. Virolainen et al. also employed Purolite A170-172 resins for the recovery of Re from sulfate solutions containing Mo and As [14]. Additionally, Purolite A170 was utilized for the selective separation of Re from copper leach solutions produced as a result of heap and stockpile leaching [15]. Rhenium recovery from uranium-bearing solutions is achievable. In a study by Zagorodnyaya et al., AN-21 (a weak base type) and Ambersep A920U (a strong base type) were employed to separate Re and U [16]. Weak resins are commonly used for rhenium extraction because they enable straightforward desorption processes, ensuring the efficient release of rhenium ions from the resin material. Laatikainen et al. explored the mechanisms of Re extraction from different solutions (nitrate, chloride, and sulfate) using Gel-type IRA-67 and WP-1 resins, highlighting the impact of adsorbent structure [17]. In another study, ZS70, a weak base resin containing a complex amine functional group, was employed for Re recovery from Cu-leach solutions [18].

An exhaustive literature survey reveals that a few numbers of research studies have been conducted on producing and improving resins for the separation and recovery of perrhenate ions (ReO_4_^−^). Xiong et al. synthesized 4-amino-1,2,4-triazole resin (4-ATR) and used it to study the static and dynamic adsorption–desorption properties of Re from a single-component solution [19]. In their 2019 study, Cyganowski and colleagues employed a microwave-assisted technique to synthesize Re(VII)-selective anion exchange resins, which were used to adsorb ReO_4_^−^ ions from synthetic acidic solutions containing Re(VII), Mo(VI), V(V), and Cu(II) [20]. In another study, they utilized the same method to synthesize three different resins using various organic solvents, namely N,N-dimethyl formamide (DMF), 1-methyl-2-pyrrolidone (NMP), and dimethyl sulfoxide (DMSO), while applying different radiation densities. The resulting resins were then tested for sorption from synthetic solutions containing either 50 or 500 mg/L of Re [21]. Guo et al. (2019) prepared coated solvent impregnated resins containing the ionic liquid Aliquat 336 (A336-CSIRs) and used them to adsorb Re from aqueous solutions with high concentrations of sulfuric acid [22]. Fathi et al. employed suspension polymerization to create three amine-functionalized resins. These resins were subsequently evaluated for their effectiveness in adsorbing perrhenate ions from binary solutions of Re and Mo [4].

An analysis of the findings from studies on functionalized resins reveals that under various process conditions, all anion exchangers containing strongly basic alkyl amines, particularly those with a cyclic structure, consistently exhibiting high sorption rates, often exceeding 99.5%. 

The present study has explored a novel, readily synthesizable, and cost-effective adsorbent for selectively extracting Re. The resin is a crosslinked polymer formed through the free radical polymerization of the diallyl dimethylammonium chloride monomer with the inclusion of a crosslinker. This technique shows great potential as a clean and effective technology for producing selective adsorbents. Following the characterization of the synthesized resin, its efficacy in selectively extracting Re (perrhenate ions) from a multi-solute solution comprising Re, molybdenum, copper, and iron was examined. Additionally, its performance was evaluated using a real solution obtained from the pressurized leaching of a molybdenite concentrate, which presented considerable complexity.

## 2. Experimental

### 2.1. Reagents and Instruments

The standard stock solutions for Re, molybdenum, copper, and iron were prepared by dissolving NH_4_ReO_4_ (Aldrich, Burlington, VT, USA), (NH_4_)_6_Mo_7_O_24_·4H_2_O (Aldrich), Copper CuSO_4_·5H_2_O (Merck, Darmstadt, Germany), and FeSO_4_·7H_2_O (Aldrich), respectively, in double-distilled water. In binary and multicomponent joint systems, the pH of the solutions was adjusted around 1 by adding sulfuric acid, and the value was held constant throughout the studied conditions. Diallyldimethylammonium chloride (DADMAC, aqueous solution 65 wt%), N,N–methylenebis acrylamide (MBA), and ammonium persulfate (APS) were provided from Aldrich.

All other reagents were of analytical grade and used without further purification. The pH was measured using a pH meter (Metrohm 827, Metrohm AG, Herisau, Switzerland). Also, the whole tests were perfumed using the Ds311 incubator shaker. Concentration of metal ions in solutions was determined using ICP-OES (Optima 7300 DV Perkin Elmer, PerkinElmer, Waltham, MA, USA), and the redox potential and pH of solutions were measured with a portable Metrohm pH and Eh meter

### 2.2. Synthesis of Crosslinked Poly DADMAC as Adsorbent

A total of 5 g of DADMAC (2 mmol) was added to a flask and its concentration was brought to 40 wt% through the addition of distilled water. In total, 0.163 g of ammonium persulfate (0.7 mmol) and 0.65 g of MBA (3 mmol) were poured into the reaction container and, after deoxygenation with nitrogen gas, the temperature was increased to 70 °C to start the polymerization reaction. The reaction continued for 12 h, and then the formed gel was precipitated in methanol. The product was washed several times with water and methanol and finally dried in a vacuum oven at 40 °C.

### 2.3. Characterization of the Synthesized Adsorbent (Poly-MADMAC)

The chemical structure and functional groups of adsorbent was characterized by Fourier-transform infrared spectroscopy (FTIR, JASCO 4100, Hachioji, Japan) within a 650 to 4000 cm^−1^ range with 4 cm^−1^ resolution. The microstructure morphology of the adsorbent was studied by FESEM (Hitachi S4160 instrument, Tokyo, Japan). The porosity of the adsorbent was surveyed by N_2_ adsorption–desorption analysis. The crystallinity property of the adsorbent was characterized using X-ray diffraction (XRD, X’PertPro, Almelo, The Netherlands) at 40 kV, 30 mA.

### 2.4. Experimental Procedure

All of the experiments were carried out under static conditions using flasks of 150 mL volume. In order to determine the amount of adsorbed metals on the anionite, the difference between the initial and final concentration of ions in the solutions was measured [23]. Equilibrium experiments were conducted at ambient conditions with specified amounts of resins (about 0.1 g) that were mixed with each of a flask series with 50 mL of ion solutions having concentrations according to varieties in natural solutions. In natural Re-bearing deposits, generally it has been accepted that through geochemical reactions and the isomorphous replacement of Mo, Re is mainly carried by molybdenite and so this element has been known as a molybdenum deposits’ co-product. On the other hand, since the major source of molybdenum (Mo) is from Porphyry-type deposits, especially the copper one with approximately 50% of annually production, accordingly, in these systems the concentrations of Mo, Cu, and Fe, iron from pyrite ores, are found higher than that of Re. Therefore, in binary and multicomponent solutions, changes were implemented to closely approximate real conditions similar to pregnant leach solutions derived from the copper processing industry. Re concentration varied within the range of 20 to 100 ppm at five intervals, while the ratios for Mo, Cu, and Fe were adjusted by factors of 5, 26, and 30, respectively. In order to reach the equilibrium state, the flasks were kept in a shaker and were stirred continuously at a shaking speed of 180 rpm for 24 h. The loading capacities of the resins were calculated using the mass balance equation below:

(1) $$q_{e}=\frac{C_{0}-C_{e}}{W}V$$

where *C_0_* and *C_e_* are the concentrations of the metal ion (mg/L) at t = 0 and the equilibrium condition, *V* is the total volume of solution (L), and *W* is the weight of dry resins (g).

### 2.5. Metal Sorption

#### 2.5.1. Synthetic Solutions

The assessment of obtained resins in Re uptake was performed in three stages. For Stage 1, five equilibrium tests were conducted with 50 mL solutions in flasks and Re concentrations: 20, 40, 50, 60, 80, and 100 ppm, to evaluate the resin’s capability in Re adsorption from the single-component systems. In the second stage, the competitive tests were carried out in the presence of Mo, and the final series was carried out using multicomponent solutions (Re-Mo-Cu-Fe). In these tests, the Re concentration was the same as in the single states (five points), while the other ions were varied as in Table 1. 

#### 2.5.2. Real Solutions

Samples of one liter each were collected at 3 h intervals from the pressurized leaching solutions of molybdenite concentrate at the Sarcheshmeh Copper Complex located in southeastern city, Sarcheshmeh, Iran, across four different work shifts. Following the collection, the samples from each shift were combined and mixed, and a representative sample of 100 mL was obtained and analyzed (Table 2).

Four sets of adsorption tests were performed under identical conditions: 0.1 g of adsorbent, 50 mL of solution, and stirring at 180 rpm, using the obtained samples. After resin loading, the process lasted for 24 h, following which the residual solution was sampled and analyzed.

## 3. Results and Discussion

### 3.1. Resin Synthesis

The polymeric adsorbent was synthesized in a crosslinked form and utilized for the selective separation of Re ions. The following tests were conducted to characterize the adsorbent: the FTIR spectrum, N_2_ adsorption–desorption test, XRD analysis, and FESEM imaging.

**Figure 1:**
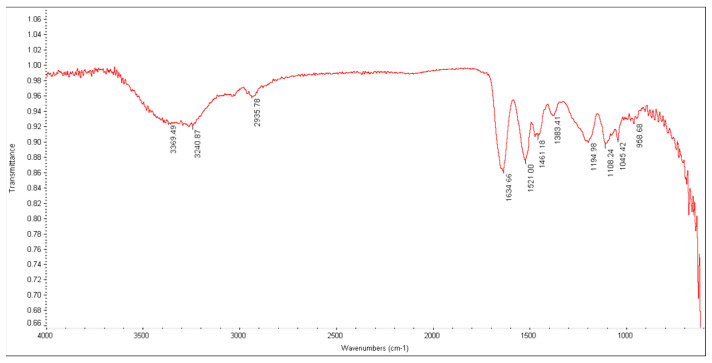
FTIR spectrum of poly−DADMAC.

The FTIR spectrum of crossliked polyDADMAC is shown in Figure 1. The characteristic peaks can be seen at 958, 1108, 1461, and 2935 cm^−1^ which were related to the stretching vibrations of quaternary ammonium groups, C–N bonds, CH_3_ groups, and CH_2_ groups, respectively. The appearance of a peak at 1634 cm^−1^ corresponded to the carbonyl groups in MBA, and the broader peak at 3369 cm^−1^ indicated surface-absorbed water.

**Figure 2:**
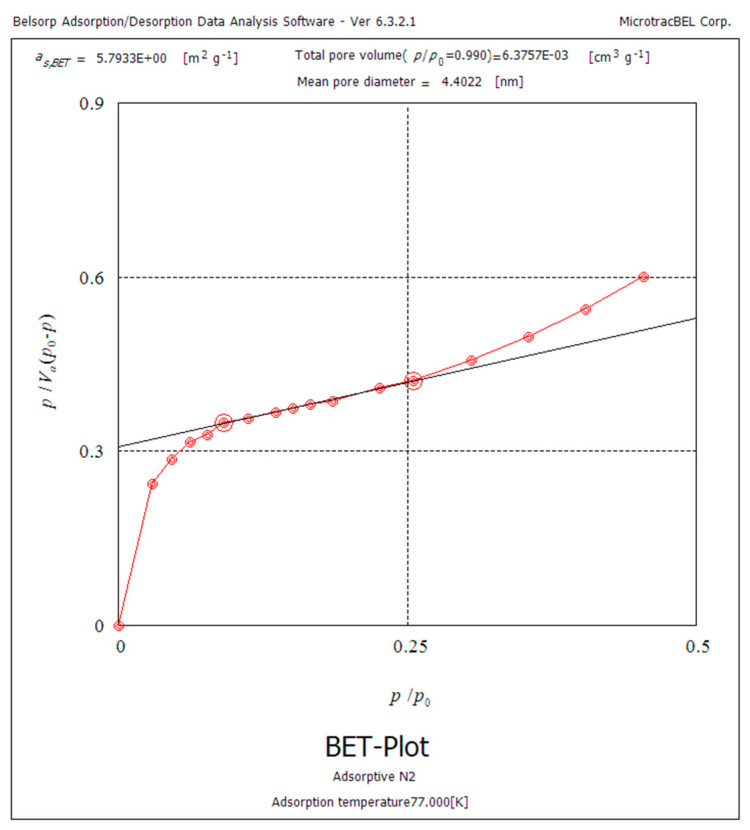
N_2_ adsorption–desorption isotherms of poly−DADMAC.

The N_2_ adsorption–desorption test was performed on the synthesized adsorbent to study the surface area, pore diameter, and pore volume. The obtained isotherm is shown in Figure 2. The analysis reveals that the adsorbent possesses a surface area of 5.7 m^2^/g, a mean pore diameter of 4.4 nm, and a pore volume of 6.4 × 10^−3^ cm^3^/g. The results indicate that the polymeric adsorbent possesses nano-sized pores and a high surface area, implying its potential as an effective adsorbent [24].

**Figure 3:**
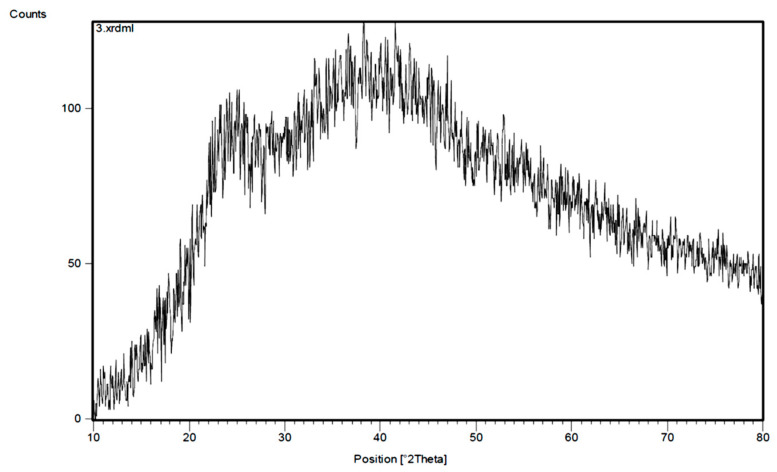
The XRD pattern of poly-DADMAC.

The XRD pattern of the polymeric adsorbent is shown in Figure 3. As can be seen in the spectrum, two broad peaks appeared at 2Ɵ = 25 and 40°, which indicate the amorphous form of the resulting polymer and also the absence of the crystalline phase in the polymer that helps to create porosity in the material and an active surface for the adsorption of desired species [25].

**Figure 4:**
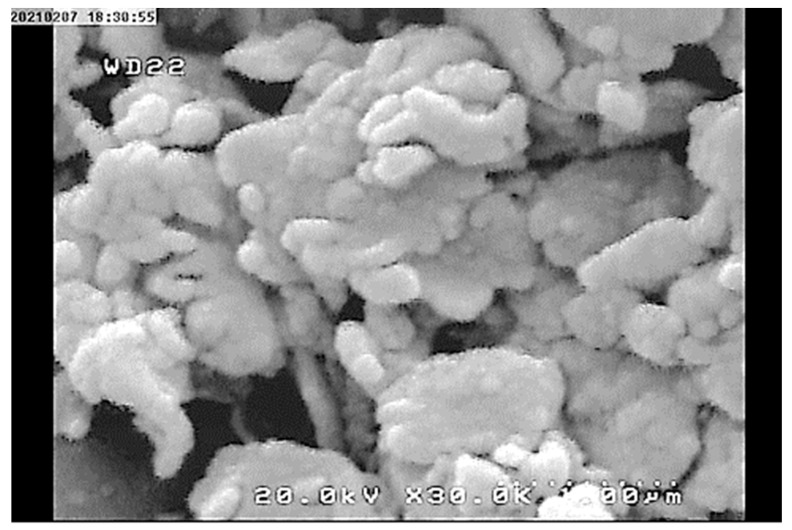
FESEM image of the poly-DADMAC.

The morphology of absorbent particles was examined using FESEM. The resulting image is depicted in Figure 4. The resulting polymer exhibits a spherical or plate-shaped particle morphology, with significant interstitial voids observed between the particles. This characteristic enhances the particle porosity and active surface area, thereby potentially improving adsorption efficiency. The porosity measurement results further validate the findings depicted in the FESEM image.

### 3.2. Resin Uptake Tests

**Figure 5:**
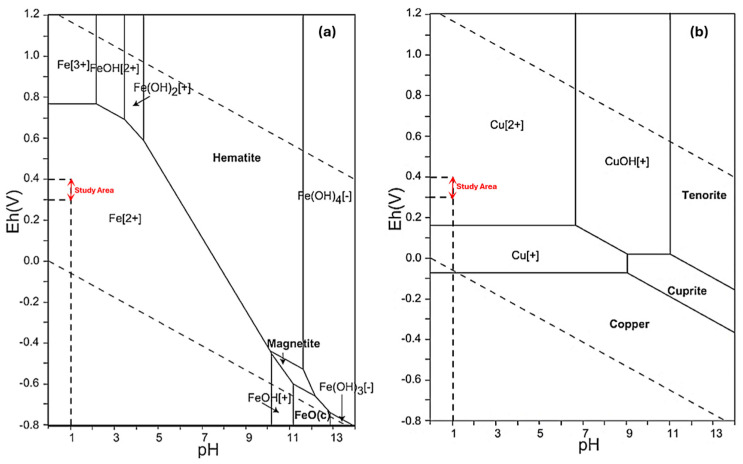
Eh-pH diagrams for the system (**a**) Fe-O-H (∑Fe = 10 −10, 298.15 K, 105 Pa) and (**b**) Cu-O-H (∑Cu = 10 −10, 298.15 K, 105 Pa). Study area: Eh = 0.3–0.4 V, pH = 1 [29].

The equilibrium tests using synthetic solutions in single (Re), binary (Re-Mo), and multicomponent systems (Re-Mo-Cu-Fe) were conducted under ambient conditions (20 °C for a 24 h). The resins’ uptakes for Re ions (*q_Re_*) from all systems were calculated with Equation (1), and the results are presented in Table 3. As seen from the data, in all single- component tests, more than 90% of perrhenate ions (ReO_4_^−^) were adsorbed by the produced resin. In these states, the resin capacity for the loading of ions was calculated over 9.2–47.2 mg/g. The amount of ions adsorbed by an adsorbate can be affected by various factors, e.g., the size of the adsorbate molecule or ion, the concentration of adsorbate, ions’ affinity to the adsorbent, the adsorbate diffusion coefficient in the bulk phase, the pore size distribution of the adsorbate, and the degree of mixing [26]. Variations in the data for Re uptake by the obtained resin in binary conditions (Re-Mo) reveal that in spite of the adsorbent high affinity for Re ions, the presence of molybdenum can lead to a drop of a about 9% in Re uptake by the resin. Previous in-depth investigations have indicated that in acidic solutions, at the pH value examined, a substantial portion of molybdenum ions are present in polyanionic forms that are notably larger than perrhenate ions [27]. Differences between the size of ions cause a large proportion of molybdenum ions to be adsorbed by the resin’s surface functional groups, and so the adsorption of perrhenate ions is constrained by competitive inhibition and their shielding effects. In multicomponent experiments, the data variations reveal that most of the ions introduced into the solution, including Cu and Fe, remained unchanged, with only a small fraction disappearing. The examination of Eh-pH diagrams for Cu and Fe (Figure 5) indicates that under the studied Eh-pH conditions (0.3 < Eh < 0.4, pH = 1), all ions exist in their cationic forms (Fe^2^⁺ and Cu^2^⁺), resulting in repulsion through the positively charged sites of the resin. The variances between the initial and equilibrium concentrations of ions can be ascribed to the electrostatic interactions among ionic and cationic species. These interactions result in the formation of complexes that can be adsorbed by resin surface groups. The effects of metal ion interferences on the adsorption capacity can be investigated by the means of *q_e_*_,*mix*_/*q_e_*, where *q_e,mix_* and *q_e_* are the sorption capacity for one metal ion in binary/multicomponent and single systems, respectively [27,28]. Based on such sorption capacities, three possible types of behavior may be exhibited: *q_e_*_,*mix*_/*q_e_* > 1, the synergism phenomenon is observed in which the sorption of target species will be promoted by the presence of other metal ions; *q_e_*_,*mix*_*/q_e_* = 1, no net interaction effect is observed; and *q_e_*_,*mix*_/*q_e_* < 1, the antagonism phenomenon is happened when the effects of the mixture are less than those of each of the individual adsorbates [27,28]. As per Table 3, in both the binary and multicomponent states, the calculated resin capacities for Re ions indicate that the variations in *q_e_*_,*mix*_/*q_e_* are consistently less than one, suggesting antagonistic effects. As stated earlier, this occurrence is due to the inhibitory influence of surface-adsorbed molybdenum ions on the binding of perrhenate ions. These ions compete with Re ions for vacant sites on the adsorbent, leading to the suppression of perrhenate ion adsorption.

### 3.3. Selectivity of the Produced Resin toward Re Ions

The affinity magnitude of the obtained resin for studied metal ions can be evaluated by the separation percentage (*r_t_*) in Equation (2).

(2) $$r_{t}=100\frac{\left(C_{0}-C_{t}\right)}{C_{0}}$$

where *C_0_* and *C_t_* are the concentrations of the metal ions at *t* = 0 and time *t*, respectively [30].

**Figure 6:**
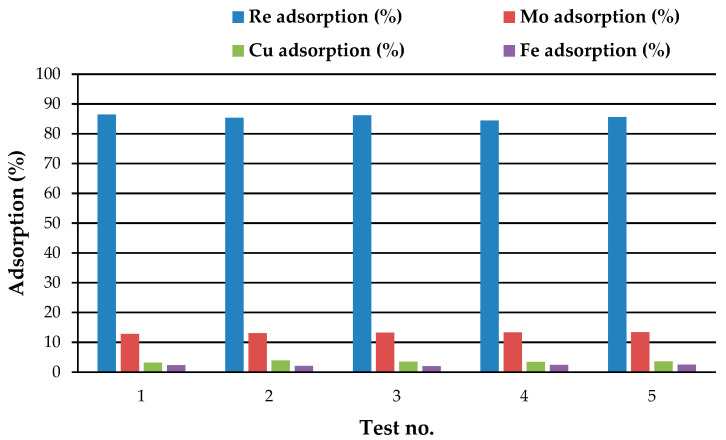
Separation percentage values in multicomponent systems, resin dosage = 0.1 mg, pH = 1.

The separation percentages of the studied elements were also calculated and are presented in Table 4. The values under binary conditions indicate that, across all scenarios considered, the adsorption of Re ions consistently exceeds that of Mo ions. Furthermore, to facilitate a better understanding of the conditions, the data obtained under multicomponent states are comparatively depicted in Figure 6. It is evident that in all tests, the separation values for Re are higher than those for other elements. This highlights the significant effectiveness of the applied function, the quaternary ammonium group in monomeric units. Employing this function and increasing the crosslinker content leads to the formation of a resin characterized by a cyclic and rigid structure, thereby enhancing its mechanical properties. This property of the resin creates steric hindrance, which hinders the effective binding of molybdenum polyanions to internal sites, resulting in their partial adsorption only by surface functional groups. On the contrary, the crosslinked structure of the resin enables sieving performance, selectively adsorbing perrhenate ions over larger molybdenum ions. Furthermore, the quaternary amine in the functional group imparts a hydrophilic nature to the resin, resulting in a high water content. The appropriate structure of the resin facilitates the diffusion of the solute through the adsorbent surface and effectively traps the small perrhenate ions from Re-bearing solutions.

### 3.4. Adsorption Tests on Real Solutions

The findings from experiments under multicomponent conditions demonstrated that the synthesized resin can efficiently purify Re under competitive circumstances. Therefore, its effectiveness was assessed using real industrial solutions. Table 4 provides information regarding the ions absorbed onto the synthesized resin. Across all four tests, it is evident that over 62% of perrhenate ions (ReO_4_^−^) were taken up by the resins. When comparing it to molybdenum adsorption, the fluctuation in results suggests that Re ions, competing with molybdenum polyanions at concentrations around 1000 times greater than Re, are absorbed by the resin around 45–50% more efficiently. This phenomenon suggests a selective separation of these two components from real solutions. It is apparent that the presence of other cationic components (Fe^2+^, Cu^2+^) does not disrupt the separation process, as they neither occupy the resin loading capacity nor contribute significantly to the loading rates. Table 5 presents a summary of Re extraction outcomes from diverse bearing solutions/conditions utilizing different resins, including both industrial and synthetic types. This allows for comprehensive comparison and analysis of the results obtained from this study and other relevant works.

**Table 4:** Results from resin uptake experiments with real solutions.

Initial Conc.				Equilibrium Conc.				Adsorption (%)			
Re (ppm)	Mo (g/L)	Fe (ppm)	Cu (ppm)	Re (ppm)	Mo (g/L)	Fe (ppm)	Cu (ppm)	Re	Mo	Fe	Cu
54.6	5.2	460.5	312.2	18.4	4.4	445.7	300.7	66.3	16	3.2	3.7
56.1	5.3	461.4	340.4	19.4	4.4	450.7	322.4	65.4	16.3	2.3	5.3
50.4	5.3	430.4	316.3	18	4.4	412.4	302.3	64.3	16.7	4.2	4.4
52.1	7.4	480.4	341.7	19.7	6.2	464.2	324.5	62.2	17.2	3.4	5.0

The structure of an anionite is crucial for determining its selection/rejection performance during the ion exchange process. In Re-Mo systems, it is understood that within a pH range of 1–10, perrhenate ions remain stable in a monomeric anion form (ReO_4_^−^), whereas molybdenum ions tend to form iso (MoO_4_^2−^, 5 < pH < 9) and/or heteropoly (Mo_8_O_26_^4−^, 1 < pH < 5) anions depending on the pH of the solution they are in contact with. In addition, the positively charged forms of molybdenum (MoO_2_^2+^, Mo_2_O_5_^2+^, and Mo_3_O_8_^2+^) can be found at pH levels below 2 [31]. Dynamic Light Scattering (DLS) analysis revealed that molybdenum polyanions are significantly larger than perrhenate ions, suggesting potential for enhancing the selective purification of Re ions from these complex solutions using specific resins’ structures. Due to the resin’s compact structure and a mean pore diameter of 4.4 nm, it appears that in real solutions, the suppression of the majority of molybdenum ions (above 82%) is also a result of the sieving mechanism of this structure. This function renders the inner functional groups of the resin inaccessible to molybdenum ions and rejects larger molybdenum molecules. In these systems, prior studies have shown that the polyanions of molybdenum are only partially adsorbed by surface functional groups, while the perrhenate anions, with a geometrical size of 0.26 nm, can still penetrate freely into the matrix (Equation (3)) [32].

–N^+^R_3(resin)_ + (ReO_4_^−^ + Mo_8_O_26_^4−^)_(aq)_ → –NR_3_ReO_4(resin)_ + Mo_8_O_26_^4−^_(aq)_ (3)

**Table 5:** Summary of Re extraction findings using different resins and solution conditions.

Bulk Solution	Elements	Resin		Efficiency	Reference
		Name	Type		
Sulfate solutions	Re-Mo	IRA-67, A172 and 170	Gel, gel, macroporous	95.3%	[14]
Molybdenite calcine leaching solution	Re-Mo	D216 and 201X7	Strong base, gel	90%	[33]
Re-Mo solution with pH 2.6–6	Re-Mo	Synthetic anion exchanger (4-amino-1,2,4-triazole)	Macroporous	βRe/Mo = 17.3	[19]
Copper heap leaching solutions	Re	Purolite A170	Macroporous	~90%	[15]
Synthetic acidic solutions (Re-Mo)	Re-Mo	Synthesized anion exchanger	Macroporous	Re uptake: 46.3 mg/g	[4]
Synthetic solutions (Re/U)	Re	Ambersep A920U	Strong base	~98.2%	[34]
Synthetic solutions (Re, U/alts)	Re	AN-21	Weakly base	33–99.2%	[16]
Different matrices (nitrate, chloride, sulfate)	Re	IRA-67, WP-1	Gel, macroporous	Aimed for modelling	[17]
Copper leaching solutions	Re	Weak base resins ZS70	Weak base	97.13%	[18]
Acidified solutions (Re(VII), Mo(VI) and V(V), Cu(II))	Re	Synthesized anion exchanger	Macroporous	Re uptake: 303 mg/g	[20]
Industrial wastewater	Re	Synthesized anion exchanger	Macroporous	99.85%	[22]
Molybdenite concentrate leaching solutions	Re	Synthesized anion exchanger	Macroporous	62.2–66.3%	This work

### 3.5. Adsorption Kinetics

**Figure 7:**
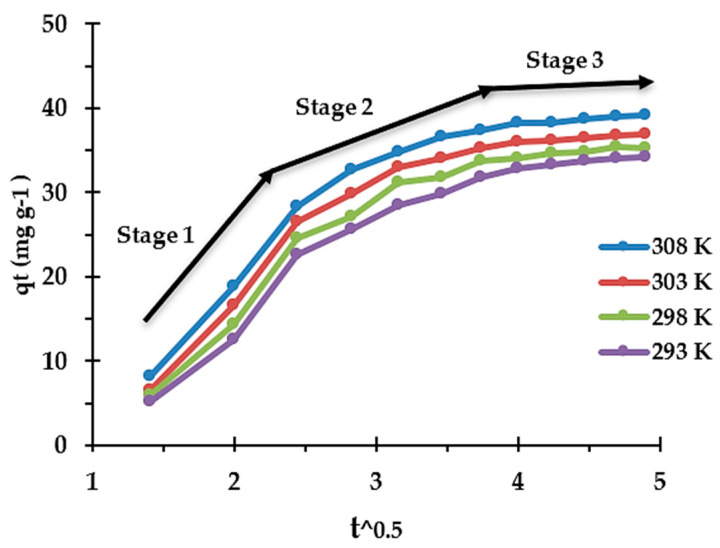
The intraparticle diffusion model to understand the adsorption kinetics of Re ions from binary (Re−Mo) systems onto the synthesized resin. C_0_: Re = 100 mg L^−1^, Mo = 500 mg L^−1^, resin dosage = 0.1 mg, pH = 1.

Examining the kinetics of liquid/solid adsorption offers insights into the rate-controlling step and the probable mechanism underlying the exchange process. Among the various models, the intra-particle diffusion equation, often referred to as the “Weber-Morris model” or the “IPD model,” stands out as one of the most commonly employed in this field (Equation (4)) [35]. In this context, five kinetic experiments were conducted using synthetic solutions under binary conditions at varying temperatures (e.g., 293, 298, 303, and 308 K), and the results were analyzed using the IPD model (Figure 7).

q_t_ = k_i_t^0.5^ (4)

where the q_t_ is the amount of adsorbate (mg g^−1^) at any time (t) and k_i_ (mg g^−1^ h^−0.5^) is the intra-particle diffusion rate constant. 

**Figure 8:**
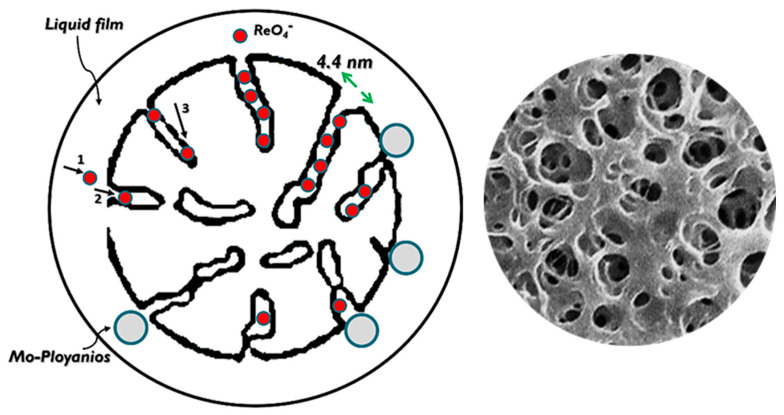
Mass transfer phenomena in the ion exchange process.

To effectively analyze the output data and acquire an understanding of the adsorption kinetics, it is crucial to comprehend the involvement of three distinct mass transfer mechanisms and their contributions to the overall process. These mechanisms include (1) external diffusion (or film diffusion), which describes the movement of the adsorbate within the liquid film surrounding the adsorbent; (2) internal diffusion (intraparticle diffusion), which refers to the migration of adsorbate within the pores of the adsorbent; and (3) adsorption onto active sites (Figure 8) [34]. Figure 6 displays the different mechanisms involved and rate constants for the absorption of Re onto the synthesized resin, where larger values correspond to faster steps, as determined by the slope of each segment. Clearly, all three steps mentioned take place during the adsorption process, each exhibiting varying kinetics. The quickest step involves the diffusion of ions through the solution to the external surface of the adsorbent boundary layer, followed by the gradual adsorption stage driven by intraparticle diffusion. As evidenced by the variations, the third part shows a notably slow rate of change, signifying the rate-limiting stage, and is connected with the final equilibrium stage. This is likely due to the shield-shaped inhibitions caused by multicharged molybdenum anions adsorbed on the resin surface, as previously demonstrated, preventing the penetration of Re ions into the bead.

## 4. Conclusions

The extraction of valuable constituents from solid residues, wastes, and effluents stands as an innovative solution propelling advancements in environmental separations. In this study, a novel cationic resin was synthesized using the free radical polymerization method for the selective adsorption and separation of perrhenate ions (ReO_4_^−^) from multicomponent solutions. Characterization tests revealed that a resin with a surface area of 5.7 m^2^/g, a pore diameter of 4.4 nm, and a pore volume of 6.4 × 10^−3^ cm^3^/g was obtained. Moreover, in the XRD pattern, the absence of a crystalline phase was observed, implying the amorphous nature of the resulting polymer, which may contribute to the creation of active surfaces with effective porosity. Following initial adsorption tests conducted on single and binary systems, two sequential test series were carried out using pre-industrial (multicomponent) and industrial solutions to evaluate the adsorbent performance. The preliminary tests under identical industrial conditions in the copper industry, at a pH of 1, and with the presence of molybdenum polyanions (Mo_8_O_26_^4−^, Mo_7_O_24_^6−^), copper, and iron ions, revealed that the resulting resin (Poly-DADMAC) exhibited a high selectivity, exceeding 80%, in adsorbing Re. This selectivity is attributed to the tight and cyclic structure derived from the quaternary ammonium group (dimethylammonium chloride) in the monomeric units. In tests with real solutions, pressurized leaching solutions of molybdenite concentrate, it was found that despite the process being affected by the substantial presence of competing ions, the synthesized resin could adsorb over 60% of Re in the solution under all investigated conditions.

## Figures and Tables

**Table 1:** The concentration of ions in competitive adsorption tests.

Ion Concentration (ppm)	Test No.				
	1	2	3	4	5
Re	20	40	60	80	100
Mo	100	200	300	400	500
Cu	520	1040	1560	2080	2600
Fe	600	1200	1800	2400	3000

**Table 2:** Chemical composition of real samples from the pressurized leaching solutions of molybdenite concentrate.

Sample/Element	Re (ppm)	Mo (g/L)	Fe (ppm)	Cu (ppm)	pH
1	56.6	5.2	460.5	312.2	0.6
2	56.1	5.3	461.4	340.4	0.5
3	50.4	5.3	430.4	316.3	0.5
4	52.1	7.4	480.4	341.6	0.5

**Table 3:** The percentage of component absorption and Re loading (q_e_) on the synthesized resin.

Adsorbate	Test No.				
	1	2	3	4	5
Re (single system) (%)	92.1	93.8	94.3	94.2	94.4
Re (binary system) (%)	87.3	86.8	86.6	85.1	86.
Re (multicomponent) (%)	86.4	85.3	86.2	84.4	85.6
Mo (binary system) (%)	13.2	13.5	13.8	13.7	13.8
Mo (multicomponent) (%)	12.8	13.1	13.2	13.3	13.4
Cu (multicomponent) (%)	3.2	3.9	3.5	3.4	3.6
Fe (multicomponent) (%)	2.3	2.1	2	2.4	2.5
q_e_ (single) (mg/g)	9.2	18.8	28.3	37.7	47
q_e,mix_ (binary) (mg/g)	8.7	17.4	26	34	43
q_e,mix_ (multicomponent) (mg/g)	8.6	17.1	25.9	33.8	43

## Data Availability

The original contributions presented in the study are included in the article, further inquiries can be directed to the corresponding author/s.
